# Microbiome and Schizophrenia

**Published:** 2019

**Authors:** Shahin Akhondzadeh

**Affiliations:** Psychiatric Research Center, Roozbeh Hospital, Tehran University of Medical Sciences, Tehran, Iran

Schizophrenia is a debilitating psychiatric disorder that contributes to a large cascade of emotional, occupational, and cognitive impairments. Treatment involves combination of psychosocial rehabilitation and pharmacotherapy. In most cases, chronic antipsychotic therapy is required to treat symptoms, avoid relapse and attenuate episode recurrence 1–3. Despite the growing number of pharmacologic agents for the treatment of schizophrenia, many patients do not adequately benefit from or tolerate currently available antipsychotics 1–3. Existing typical and atypical antipsychotic medications are relatively equally effective in treating what are known as the positive symptoms of schizophrenia. What has been prominently lacking, however, is an agent that also treats the negative symptoms as well as substantial cognitive impairment 1–3. Despite growing numbers of antipsychotic drugs for the treatment of schizophrenia, the management of this disorder remains to be a major challenge. Therefore, there is a need to find new strategies to improve treatment plans for schizophrenia patients. New studies have found that people with schizophrenia have differences in their gut biomes compared to people without the mental disorder 4,5. The researchers found a smaller subset of bacteria that were clearly different between schizophrenia patients and those without the disorder. They report that when they introduced samples of the subset from the schizophrenia patients into the biomes of healthy mice, the mice displayed behavior changes 6,7. The researchers claim that their results show that people with schizophrenia have differences in their gut biomes and that those differences may be associated with schizophrenia symptoms. They suggest that certain bacteria in the biome may be associated with schizophrenia-related symptoms due to interactions with microbiota gut-brain amino acids, and possibly lipid metabolic pathways. In conclusion, researchers have started to find interesting links between the naturally occurring bacteria that live in our guts, and things we’ve traditionally attributed to the brain. Things like our mood, feelings, and even thoughts 8.
